# Culture Model for Non-human Primate Choroid Plexus

**DOI:** 10.3389/fncel.2019.00396

**Published:** 2019-08-28

**Authors:** Elizabeth C. Delery, Andrew G. MacLean

**Affiliations:** ^1^Division of Comparative Pathology, Tulane National Primate Research Center, Covington, LA, United States; ^2^Tulane Program in Biomedical Sciences, New Orleans, LA, United States; ^3^Department of Microbiology and Immunology, Tulane Medical School, New Orleans, LA, United States; ^4^Tulane Brain Institute, New Orleans, LA, United States; ^5^Tulane Center for Aging, New Orleans, LA, United States

**Keywords:** choroid plexus, epithelial cell, cell culture, rhesus macaque, aging, infectious disease

## Abstract

While there are murine and rat choroid plexus epithelial cell cultures, a translationally relevant model for choroid plexus activation and function is still lacking. The rhesus macaque is the gold standard for modeling viral infection and activation of CNS, including HIV-associated neurocognitive disorders. We have developed a rhesus macaque choroid plexus epithelial cell culture model which we believe to be suitable for studies of inflammation associated with viral infection of the CNS. Epithelial morphology and function were assessed using vimentin, phalloidin, the tight junction protein zonula-occludens-1 (ZO-1), and focal adhesion kinase (FAK). Choroid plexus epithelial cell type was confirmed using immunofluorescence with two proteins highly expressed in the choroid plexus: transthyretin and α-klotho. Finally, barrier properties of the model were monitored using pro- and anti-inflammatory mediators (TNF-α, the TLR2 agonist PamCys3K, and dexamethasone). When pro-inflammatory TNF-α was added to the xCelligence wells, there was a decrease in barrier function, which decreased in a step-wise fashion with each additional administration. This barrier function was repaired upon addition of the steroid dexamethasone. The TLR2 agonist PAM3CysK increased barrier functions in TNF-α treated wells. We have presented a model of the blood-CSF barrier that will allow study into pro- and anti-inflammatory conditions in the brain, while simultaneously measuring real time changes to epithelial cells.

## Introduction

The choroid plexus, comprising the blood-cerebrospinal fluid (CSF) barrier, lines the lateral, 3rd and 4th ventricles of the brain (Zheng and Zhao, 2002; Monnot and Zheng, 2013). It is responsible for secreting CSF, allowing the passive diffusion of water and oxygen into the brain, and facilitating the active transport of glucose and larger particles into the brain (Zheng and Zhao, 2002; Monnot and Zheng, 2013). It has low pinocytotic activity and also contains selective transporters including multidrug resistance proteins that are responsible for allowing drugs into, and out of, the brain (Rao et al., 1999; Varatharajan and Thomas, 2009; Schwerk et al., 2015). The choroid plexus also has a unique double-layer structure of relatively leaky fenestrated capillary endothelium and tight junction dense epithelium separated by a narrow space devoid of astrocytes and neurons (Liddelow, 2015). There are also unique populations of macrophages that line the endothelial blood vessels and choroid plexus resident macrophages (Bragg et al., 2002; Kim et al., 2006; Delery and Maclean, 2018).

The choroid plexus is a site of monocyte transmigration during normal immune surveillance and has been proposed to be a site of HIV entry into the CNS (Falangola et al., 1995; Maslin et al., 2005), and a possible reservoir site (Petito et al., 1999; Meeker et al., 2012). Transmigration of monocytes and leukocytes is also increased after stroke (Ge et al., 2017; Xiang et al., 2017), during bacterial (Canova et al., 2006; Schwerk et al., 2015) and viral infections (Feuer et al., 2003; Tabor-Godwin et al., 2010). The choroid plexus is also critical for brain inflammaging – the accelerated aging observed in chronic inflammation. Choroid plexus epithelial cells contain the protein α-klotho (German et al., 2012; Zhu et al., 2018), which acts as an “anti-aging hormone” (Kuro-o et al., 1997; Takahashi et al., 2000). Decreases in α-klotho have been linked to numerous neurodegenerative disorders including multiple sclerosis and Alzheimer’s Disease (Torbus-Paluszczak et al., 2018).

Several rodent models of the choroid plexus exist, including immortalized rat Z310 and TR-CSFB, as well as primary mouse models (Kläs et al., 2010; Menheniott et al., 2010; Monnot and Zheng, 2013; Barkho and Monuki, 2015). Porcine choroid plexus epithelial cell lines have been used to study bacterial and drug infection of the brain (Haselbach et al., 2001; Angelow et al., 2004; Schwerk et al., 2011). However, the murine models have reported difficulty in proliferation in culture, the immortalized rat cultures have altered tight junction proteins compared to primary rat cultures, and none of the models is useful for studies of HIV infection (Angelow et al., 2004; Abel, 2009; Barkho and Monuki, 2015; Beck et al., 2017). To our knowledge, there currently is not a model that facilitates real-time measures of barrier function using label-free measurements. As viruses and viral proteins are likely to have effects over different time periods, such a model would be useful. Combined, there is a critical need for a translational choroid plexus model for neurovirology, neurodegeneration, and inflammaging studies. As the rhesus macaque is the gold standard animal model of studying viral pathogenesis *in vivo*, we designed a primary rhesus macaque choroid plexus epithelial cell culture.

Beginning with published methodologies for choroid plexus epithelium (Zheng and Zhao, 2002; Monnot and Zheng, 2013) and our previously established microvascular brain endothelial cells isolation protocol (MacLean et al., 2001, 2002; Ivey et al., 2009b; Sansing et al., 2012), we designed and tested the following protocol for isolation, culture, and characterization of choroid plexus epithelial cells.

## Materials and Methods

### Primary Cell Culture Protocol

Rat tail collagen (Sigma-Aldrich, St. Loius, MO, United States) was dissolved in 0.1 M acetic acid to create the collagen-coated plates. Twenty-four hours prior to necropsy, 100 μL/well of the collagen solution was added to a 12 well plate. Gelling of collagen was induced by addition and changing M199 until the media did not change color. Plates were incubated at 37° and 5% CO_2_ overnight, with media on top in order to prevent the collagen from drying out.

Choroid plexuses from juvenile (1–5-year-old) Indian-origin Rhesus macaques were obtained at investigator-initiated necropsy at Tulane National Primate Research Center (TNPRC). Animals were housed and humanely euthanized according to standards set forth by the Office of Laboratory Animal Welfare (OLAW) and TNPRC’s Institutional Animal Care and Use Committee (IACUC). A veterinary pathologist from TNPRC collected choroid plexus from the lateral, 3rd and 4th ventricles into PBS containing antibiotics/antimycotics (unless otherwise stated, all media was obtained from Gibco).

**FIGURE 1:**
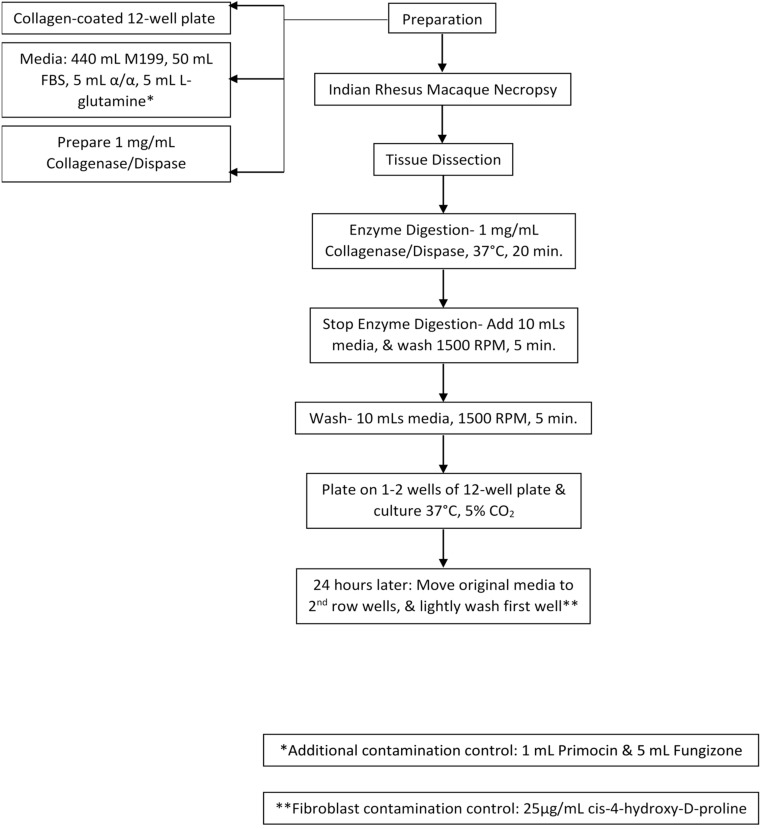
Cell Culture Model Schematic. Collagen-coated plates, cell culture media, and digestion media were prepared. After necropsy, choroid plexus was collected and dissected. Next, they were treated with enzyme digestion for 20 min, washed, and plated. 24-h later media was re-plated. Cells were then treated with *cis-*4-hydroxy-D-proline to control for fibroblast contamination.

Using tweezers and a scalpel, the choroid plexus was minced and incubated with 2 mL collagenase/dispase (1 mg/ml, Roche, Indianapolis, IN, United States) at 37°C and 5% CO_2_ for 15–20 min. The digested tissue was centrifuged and washed twice with gentle trituration in 10 mL of complete media (450 ml M199, 50 mL exosome-depleted fetal bovine serum, 5 ml antibiotics/antimycotics, 1 mL Primocin (Invivogen) and 5 mL L-glutamine). Dissociated cells were resuspended in complete media and gently plated on top of the collagen gel (∼2 mL/well) and incubated at 37°C and 5% CO_2_. Any contaminating fibroblasts in the primary cultures were inhibited with 3–5 days incubation with 25 μg/ml *cis-*4-hydroxy-D-proline in the complete media (Sigma-Aldrich, St. Louis, MO, United States). Reference Figure 1 for a step-by-step visualization of the protocol.

As cells approached confluence, they were sub-cultured using standard techniques (MacLean et al., 2001, 2002; Ivey et al., 2009b; Sansing et al., 2012). Either collagen or gelatin was suitable for coating culture substrates beyond primary culture. In brief, cells were washed twice sequentially with PBS, then PBS-versene before trypsin-versene was added. Trypsin was stopped with complete media, and cells were washed and centrifuged twice prior to plating on the new plate or flask.

### Immunocytochemical Confirmation of Cell Type

Immunocytochemistry was performed to positively identify choroid plexus epithelial cells using antibodies to transthyretin, vimentin, phalloidin, zonula-occludens-1 (ZO-1), focal adhesion kinase (FAK), and α-klotho. Isotype controls were used to confirm positivity. Transthyretin has been reported to be a unique marker of choroid plexus epithelial cells (Emerich et al., 2006; Monnot and Zheng, 2013; Lun et al., 2015; Spector et al., 2015). Both vimentin and phalloidin were used in conjunction with transthyretin to confirm cellular morphology (MacLean et al., 2005; Renner et al., 2013; Tavazzi et al., 2014). Choroid plexus epithelial cells form a barrier *in vivo*, and express the tight junction protein Zonula-occludens-1 (ZO-1) (Howarth and Stevenson, 1995; Emerich et al., 2006; Szmydynger-Chodobska et al., 2007; Ivey et al., 2009a, b). FAK is expressed in epithelial cells (Playford et al., 2008), and is intimately associated with both transthyretin (Zawiślak et al., 2017) and ZO-1 (Ivey et al., 2009b). Within the CNS, α-klotho expression is highest in the choroid plexus, thus making it another marker to confirm cell phenotype (Kuro-o et al., 1997; Takahashi et al., 2000; German et al., 2012; Clinton et al., 2013). We ruled out contamination with other CNS cell types using the following antibodies: CD163 (macrophage lineage cells), allograft inflammatory factor 1 (microglia and macrophages), glial fibrillary acidic protein (astrocytes).

The media was pipetted off the coverslips, and cells were blocked for 15–60 min with 1% paraformaldehyde. Cells were permeabilized in PBS-FSG-Triton ×100 for 1 h before primary antibody was added at the previously reported concentrations. Cells were then washed with PBS-FSG for 5 × 5 min before secondary antibody was added for 1 h. For multiple stains the primary and secondary process was repeated. Coverslips were then placed on glass slides with DAPI mounting media.

### Real-Time Monitoring of CSF Barrier Model

Cells were plated at a concentration of 10,000 cells/well on gelatin-coated xCELLigence 16 well E-plates (Acea Biosciences, San Diego, CA, United States). Cell indices were recorded continuously until plateaus were reached, indicating confluence (Sansing et al., 2012; Kumar et al., 2018). Wells were treated with one or a combination of: TNFα (100 U/ml), the TLR2 agonist Pam3CysK (1 μg/ml), or dexamethasone (1 μM, freshly prepared from a 10 mM stock dissolved in DMSO). Multiple controls were used, including media-only wells, and single-treated wells in the experiments where a combination of treatments were used. Four wells were used for each condition, and they were averaged together to produce our results using the inbuilt software. Error bars are ± standard deviation. Significance of increased or decreased impedance was calculated using Prism v8.2 using paired two-tailed *t*-tests.

## Results

### Cell Culture

**FIGURE 2:**
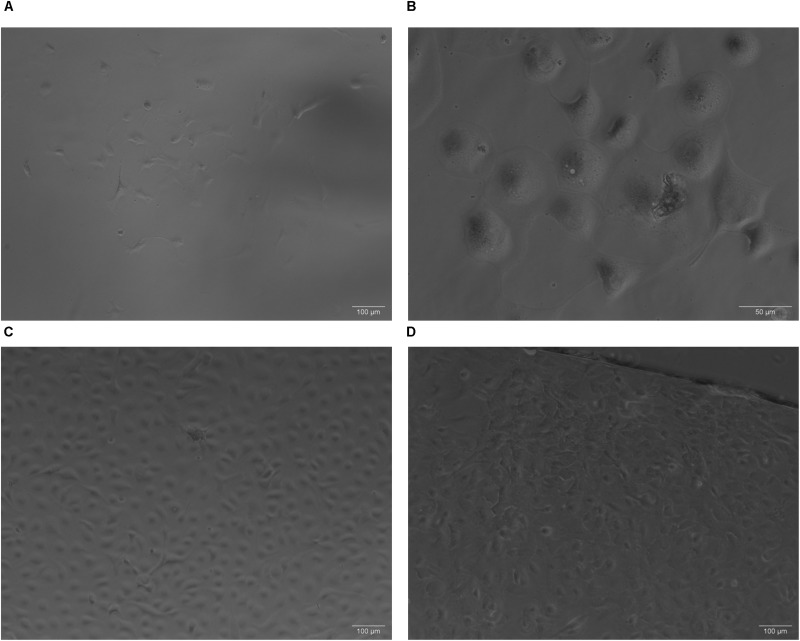
Phase contrast images of primary cultures on collagen. **(A)** On initial plating, choroid plexus-derived cells had a circular morphology that became a mixed morphology about 1 week later (**A**, Day 10, 10× magnification). Within 2–3 weeks aggregates of cells start forming, with cuboidal/epithelial morphology (**B**, Day 22, 32× magnification). After 3–4 weeks, monolayers were evident (**C** Day 22, 10× magnification). Some cultures from the same animal grew faster than others depending on initial seeding density. Cells subcultured onto collagen coated coverslips reached confluence in approximately 10 days (**D** 10× magnification).

On initial plating, choroid plexus-derived cells had a circular morphology that became a mixed morphology after about 7 days (Figure 2A). By ∼14 days post isolation, there is evidence of aggregates of cells forming, with cuboidal/epithelial morphology (Figure 2B). After 4–5 weeks, monolayers were evident (Figure 2C). Cells were sub-cultured ∼1:4 after monolayer formation and reached confluence again after 2–3 weeks when grown on collagen coated coverslips (Figure 2D). Although collagen appeared to improve the initial plating of primary choroid plexus cultures, choroid plexus epithelial cells grew as monolayers on either gelatin or collagen coated plates (not shown).

### Immunocytochemical Confirmation of Choroid Plexus Epithelial Phenotype

**FIGURE 3:**
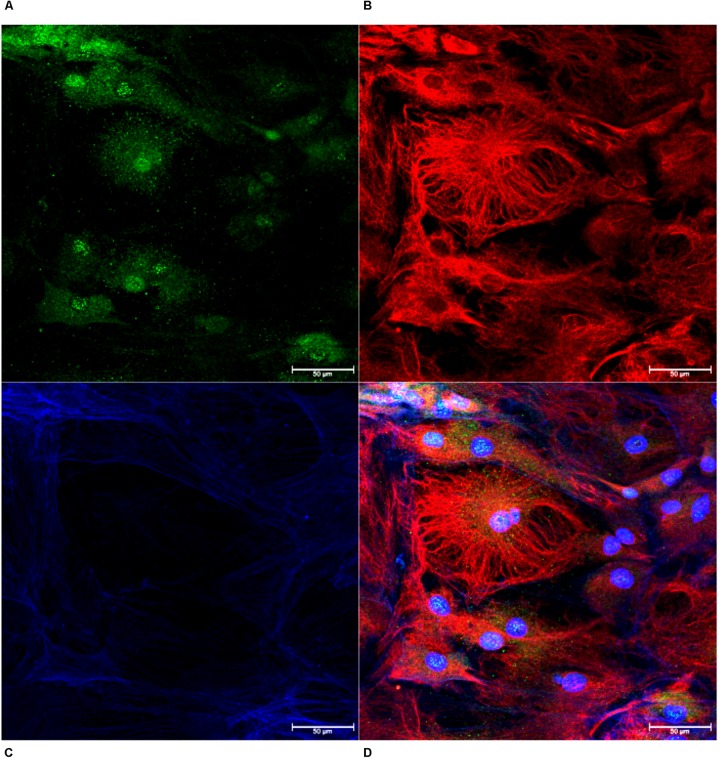
Confirmation of Choroid Plexus Epithelial Cells. Monolayers of cells were confirmed as choroid plexus using antibodies against transthyretin (**A**, green), vimentin (**B**, red) and phalloidin (**C**, blue). The combined image demonstrates cells positive for transthyretin with cuboidal cell structure, confirming epithelial cells extracted from the choroid plexus (**D**, 63×).

Monolayers of choroid plexus epithelial cells grown on glass coverslips were immunopositive for transthyretin (Figure 3A), vimentin (Figure 3B) and phalloidin (Figure 3C). Thus, cells with cuboidal and cuboidal-like morphologies that expressed transthyretin were identified as choroid plexus epithelial cells (Figure 3D). Cells were also negative for CD163, a macrophage marker (Kim et al., 2006; Borda et al., 2008; Fischer-Smith et al., 2008), Iba-1, a macrophage/microglial marker (Renner et al., 2012), and GFAP, an astrocyte marker (MacLean et al., 2002; Renner et al., 2013; Lee et al., 2014) (not shown).

**FIGURE 4:**
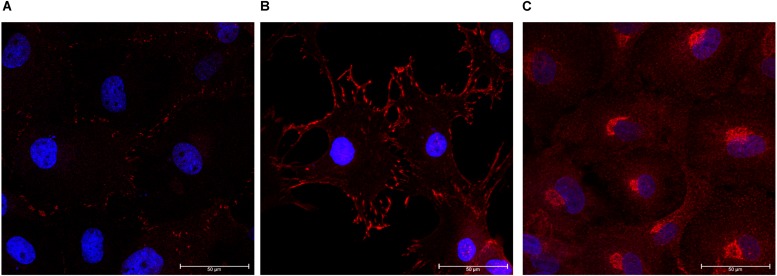
Formation of tight junctions, cellular adhesions, and presence of anti-aging hormone. The choroid plexus cells expressed the tight junction protein zonula-occludens (**A**, ZO-1 in red; DAPI in blue 63× magnification). Cells were also positive for focal adhesion kinase (**B**, 63×). Consistent with previous reports, the anti-aging protein α-klotho was found in highest accumulation adjacent to the nuclei (**C**, 63× magnification).

Expression of zonula occludens-1 (ZO-1) and FAK was confirmed in monolayer cultures on glass coverslips. Cells were positive for ZO-1 indicating the presence of tight junctions (Figure 4A). We determined that the choroid plexus epithelial cells were immunopositive for FAK, which indicates cell-cell adhesions (Figure 4B). The choroid plexus cells were also immunopositive for α-klotho, in a primarily perinuclear manner (German et al., 2012; Figure 4C).

### Real-Time Model of Barrier Function

**FIGURE 5:**
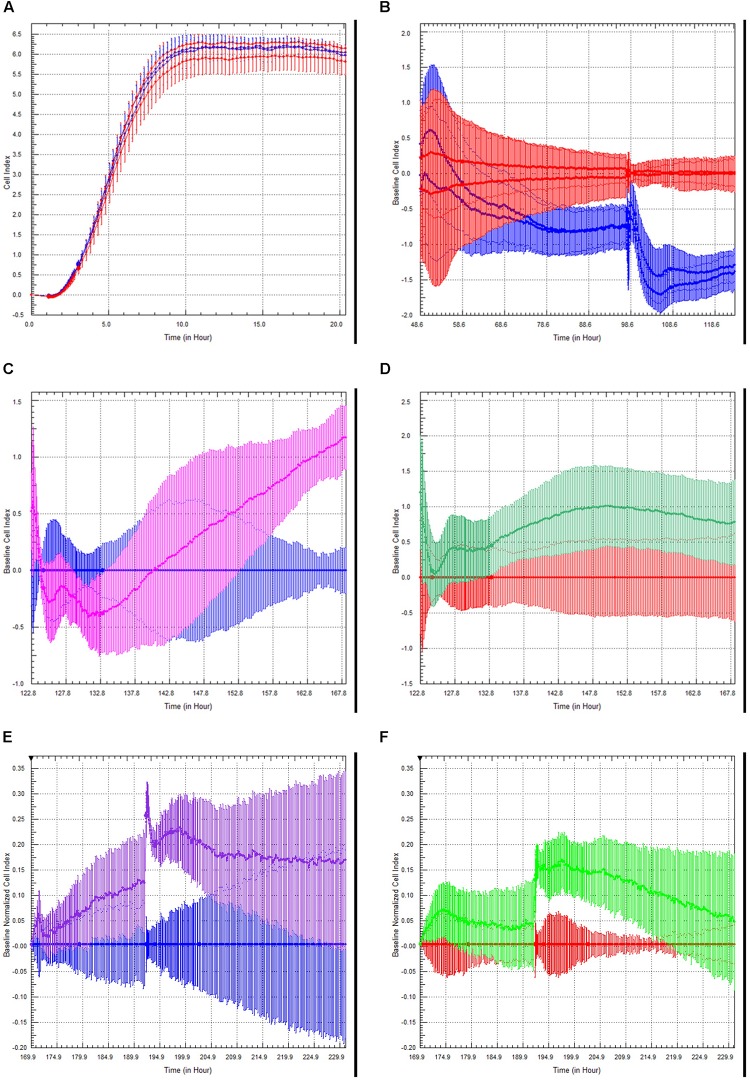
Choroid Plexus Epithelial Cell Growth, Barrier function, and modulation of barrier function by TLR2 agonists and steroids. Choroid plexus epithelial cells plated on E-plates produced a characteristic growth phase and plateau indicating monolayer formation **(A)**. After the addition of TNFα (100 U/mL), there was an initial increase in cell index peak followed by a sustained decrease (red = M199 media, blue = TNFα) **(B)**. This effect was amplified with a second challenge of TNFα. There was an unexpected increase in cell index following treatment with the TLR2 agonist Pam3CysK in cells primed with TNFα (blue = TNFα, pink = TNFα followed by PamCys3K) **(C)**. There was considerably less effect in naïve choroid plexus cells treated with PamCys3K versus media controls (red = media only, green = PamCys3K) **(D)**. In separate experiments, cells were treated the cells with anti-inflammatory steroid dexamethasone after priming with TNFα. As anticipated, dexamethasone treatment increased the cell index compared to TNFα only treated cells (blue = choroid plexus cells + TNFα, purple = choroid plexus cells + TNFα + dexamethasone) **(E)**. However, dexamethasone added to naive cells only induced a very modest increase in cell index compared to media alone (red = media only, green = naïve cells + dexamethasone) **(F)**.

Choroid plexus epithelial cells at passage 3–4 were plated on xCELLigence E-plates, and allowed to adhere and spread overnight (Figure 5A). Increased Cell Index is indicative of increased impedance (Sansing et al., 2012). Once traces had reached a plateau, we modeled neuroinflammation by adding TNFα (100 U/ml) to half the wells (blue traces). This induced an initial slight increase in cell index, followed by a steady, sustained decrease (Figure 5B, red = M199, blue = TNFα). A second administration of TNFα, 48 h later, induced a steeper and significant decrease in cell index (*p* < 0.005, *n* = 4). This indicates that TNFα induces barrier dysfunction of the rhesus choroid plexus epithelial cells.

Toll-like receptor (TLR2) is expressed on the choroid plexus of rhesus macaques (Delery et al., under review). We examined the effects of the TLR2 agonist PAM3CysK on choroid plexus barrier function. The greatest effect of PamCys3K was on cells pre-treated with TNFα, where we observed a sharp increase in cell index beginning approximately 10 h after incubation with PAM3CysK (Figure 5C, blue = TNFα followed by M199, pink = TNFα followed by PamCys3K, *p* < 0.001, *n* = 4). PamCys3K also increased cell index when added to naïve control choroid plexus epithelial cells, compared to a baseline of M199 treatment; however, this increase in cell index was neither significant nor sustained (Figure 5D, red = M199, green = PamCys3K, *p* = 0.597).

Finally, we examined the effect of the anti-inflammatory steroid dexamethasone on the barrier function. Following administration of 100 U/ml TNFα, the cultures were treated with dexamethasone (1 μM). Compared to TNFα treated cells only, the TNFα + dexamethasone treated cells saw a greater increase in cell index, with this effect increasing with each subsequent dose (Figure 5E, blue = TNFα only, purple = TNFα followed by dexamethasone). Dexamethasone only modestly increased the cell index in naive choroid plexus monolayers, compared to control cells (Figure 5F, red = M199, green = dexamethasone).

## Discussion

While the choroid plexus is a critical brain structure that allows for the selective trafficking of immune cells into the brain during normal surveillance (Williams and Hickey, 1995), it is also an understudied viral entry point. Due to the highly fenestrated nature of the endothelial cells of the choroid plexus, it is quite possible that the choroid plexus could act as a “back-door” for HIV entry into the brain. As HIV does not have a translationally relevant small animal model, we believed it was critical to develop a choroid plexus cell culture model in a species that does allow studies of HIV neuropathology: the rhesus macaque (Ivey et al., 2009a). We believe this model will also be useful for the study of accelerated aging or “inflammaging,” as the choroid plexus is the main source of α-klotho, the “anti-aging hormone,” in the brain (Kuro-o et al., 1997; Takahashi et al., 2000; German et al., 2012).

This cell culture model of the choroid plexus was verified by the presence of transthyretin, vimentin, phalloidin, ZO-1, FAK, and α-klotho. It is well established that transthyretin is a unique marker of choroid plexus epithelial cells (Zheng and Zhao, 2002; Baehr et al., 2006; Monnot and Zheng, 2013). By performing double- and triple-labeled immunocytochemistry with vimentin and phalloidin, both structural proteins (MacLean et al., 2005; Renner et al., 2013; Tavazzi et al., 2014), we determined the cuboidal morphology of the transthyretin positive cells. The cells also expressed the tight-junction protein, ZO-1, which is also of functional importance to choroid plexus barrier function (Howarth and Stevenson, 1995; Emerich et al., 2006; Szmydynger-Chodobska et al., 2007; Ivey et al., 2009b). Lastly, positive staining for the anti-aging protein α-klotho, further bolster this cell culture model of choroid plexus barrier (Kuro-o et al., 1997; Takahashi et al., 2000; German et al., 2012). These results were replicated in cultures derived from five animals.

Collagen-coated plates permitted selective attachment and growth of epithelial cells and allowed us to change the media and wash off debris with no adverse effects, including removal of poorly adhered cells, resulting in minimal-to-no fibroblast contamination. While growth was slower than other CNS cell types cultured in our labs, with this model taking about 6–8 weeks to reach a level of confluence to subculture, once established the choroid plexus epithelial cells were stable for at least five sub-cultures as confirmed by immunofluorescence with the aforementioned phenotypic markers.

After sub-culturing onto xCelligence plates we monitored the barrier properties in real-time. TNF-α appeared to have a cumulative effect on the choroid plexus epithelial cells (Figure 5B), as cell index was subsequently lower after sequential TNFα challenges. This has interesting implications for multiple inflammatory challenges for overall brain health and repair both *in vitro* (Sansing et al., 2012) and *in vivo* (Peterson et al., 2019).

We have previously shown increased TLR2 expression in the CNS in response to SIV, among other neuroinflammatory conditions (Lee et al., 2013a, b, 2014, 2015, 2016; Snook et al., 2014; Lee and MacLean, 2015; Inglis et al., 2016; Robillard et al., 2016; Chiu et al., 2019). Stimulation with TLR2 agonists would decrease BBB function both directly (Zhu et al., 2018), and indirectly, by activating astrocytes to secrete proinflammatory cytokines (Min et al., 2015) and induce leukocyte infiltration (Min et al., 2015). Indeed, increased TLR2 expression in parenchymal CNS cells has been linked to inflammaging (Wilhelm et al., 2017). In the current study, the TLR2 agonist PamCys3K increased cell index over time (Figures 5C,D). This effect was greatest in cells previously challenged with TNFα (Figure 5C), however, PamCys3K administration still increased cell index in naive choroid plexus epithelial cells (Figure 5D). The apparent dichotomy in our study is therefore most likely due to the epithelial nature of the cultured choroid plexus cells, rather than endothelial cells. Indeed, TLR2 increases barrier properties of epithelial cells in culture (Ragupathy et al., 2014). Our ongoing studies indicate that TLR2 is present in the choroid plexus epithelial cells of rhesus macaques, and this is lower in animals infected with SIV (Delery, under review).

As anticipated, the anti-inflammatory steroid dexamethasone increased cell index, and was even able to return TNFα treated cells close to normal cell index (Figures 5E,F). The effect was more pronounced in cells primed with TNFα than naïve choroid plexus cells, indicating a relatively intact barrier to begin with.

We have presented a model of the blood-CSF barrier that will allow study into pro- and anti-inflammatory conditions in the brain, while simultaneously measuring real time changes to choroid plexus epithelial cells. This rhesus macaque model could also allow us to study viral infections *ex vivo*. We propose using this model examine how multiple inflammatory challenges, or long-term exposure to anti-retroviral drugs could lead to early inflammaging.

## Data Availability

All datasets generated for this study are included in the manuscript and/or, the supplementary files.

## Ethics Statement

Animal Subjects: The animal study was reviewed and approved by the Tulane University IACUC.

## Author Contributions

ED performed the cell cultures under the guidance of AM who oversaw all procedures. ED and AM contributed to the writing of this manuscript.

## Conflict of Interest Statement

The authors declare that the research was conducted in the absence of any commercial or financial relationships that could be construed as a potential conflict of interest.
